# Vertebral Body Erosion and Subsequent Back Pain Secondary to a Vena Cava Filter

**DOI:** 10.7759/cureus.250

**Published:** 2015-02-20

**Authors:** William Newman, Nathan T Zwagerman, Peter C Gerszten

**Affiliations:** 1 Neurological Surgery, Department of Neurological Surgery, University of Pittsburgh Medical Center; 2 Neurological Surgery, University of Pittsburgh; 3 University of Pittsburgh

**Keywords:** inferior vena cava filter, vertebral body erosion

## Abstract

Background: A patient with multiple open lumbar procedures, the most recent of which was complicated by symptomatic pulmonary embolus, underwent placement of an inferior vena cava filter (IVCF). Two years after placement, she developed low back pain with radicular symptoms. CT of the lumbar spine demonstrated vertebral body erosion from the IVCF strut. In this brief report, we describe L2 vertebral body erosion, radiographic findings, and a brief review of the literature.

Objective: To describe radiographic findings of vertebral body erosion from IVCF as well as review the literature regarding this and other complications of IVCF placement.

Methods: Retrospective, single patient chart review.

Results: The patient's IVCF was successfully removed without complication. The pain and radicular symptoms resolved by one month follow-up.

Conclusions: Based on the literature, most IVCFs with evidence of bony erosion are removed except when patient comorbidities, goals of care, or a complete absence of symptoms make removal inadvisable.

## Introduction

A patient with multiple open lumbar procedures, the most recent of which was complicated by symptomatic pulmonary embolus, underwent placement of an inferior vena cava filter (IVCF). Two years after placement, she developed low back pain. CT of the lumbar spine demonstrated vertebral body erosion from the IVCF strut. In this brief report, we describe L2 vertebral body erosion, radiographic findings, and a brief review of the literature.

## Case presentation

The University of Pittsburgh Institutional Review Board issued approval IRB # PRO08120394 for this case report. Informed patient consent was obtained for her treatment.

**Figure 1:**
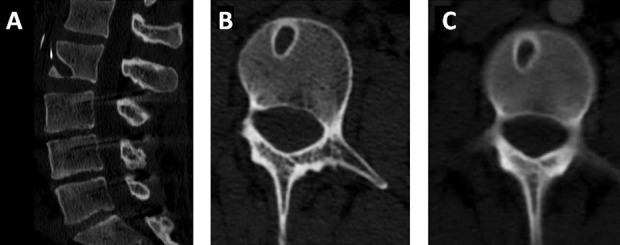
Erosive changes of the L2 vertebral body CT imaging demonstrates erosive changes of the L2 vertebral body with a prong of the IVCF violating the outer cortex (Figure 1A).

A patient had undergone several open lumbar procedures, including pedicle screw fixation. The last surgery was complicated by a symptomatic pulmonary embolus. At that time, the patient underwent the uneventful placement of an inferior vena cava filter (IVCF). Two years after placement of the IVCF, the patient began having lower back pain with intermittent right flank pain. Four months later, the patient developed symptoms of urinary retention. The work-up for these symptoms included a CT of the lumbar spine. CT imaging demonstrated erosive changes of the L2 vertebral body with a prong of the IVCF violating the outer cortex (Figure 1A).  

A vascular surgery consultation recommended endovascular removal of the IVCF, which was performed without complication through an internal jugular approach. By one month follow-up, the new pain symptoms had resolved. Post-removal imaging demonstrated stable changes of the L2 vertebral body (Figure 1B, 1C).

## Discussion

Inferior vena cava filter (IVCF) placement in patients at risk for thromboembolic events and contraindications to systemic anticoagulation is common. The literature demonstrates an overall complication rate of 10% [1], with rates of IVCF migration and fracture of less than 1.8% at six months and less than 4.3% at 12 months [2]. While complications involving the vertebral body are uncommon, these are not an unknown complication of IVCF placement [3-5]. However, vertebral body involvement alone is rarely seen. More frequently, such vertebral body involvement is associated with vascular complications, such as a pseudoaneurysm [6], retroperitoneal hematoma [7], or hematemesis from esophageal erosion [5]. The time to presentation amongst these case reports ranges from two days in the case of acute vascular complications [7] to 15 years for asymptomatic vertebral body erosion [4]. In our patient, the vertebral body erosion was found two years after IVCF placement due to the onset of symptoms of back pain and urinary retention, both of which improved after removal.

Despite evidence of bony erosion, these IVCF often are not removed secondarily to overall patient comorbidities, goals of care, or lack of symptoms [3-4, 8].  However, given the potential for adverse vascular and gastrointestinal complications as well as IVCF migration [2], removal of a no longer necessary IVCF should be attempted.

## Conclusions

Based on the literature, most IVCFs with evidence of bony erosion are removed except when patient comorbidities, goals of care, or a complete absence of symptoms make removal inadvisable.
